# Supplementary figures and images for: Multimodal imaging and functional analysis of the chick NMDA retinal damage model
Source: PLoS One. 2021 Sep 7;16(9):e0257148. doi: 10.1371/journal.pone.0257148 (PMC8423281; doi:10.1371/journal.pone.0257148)

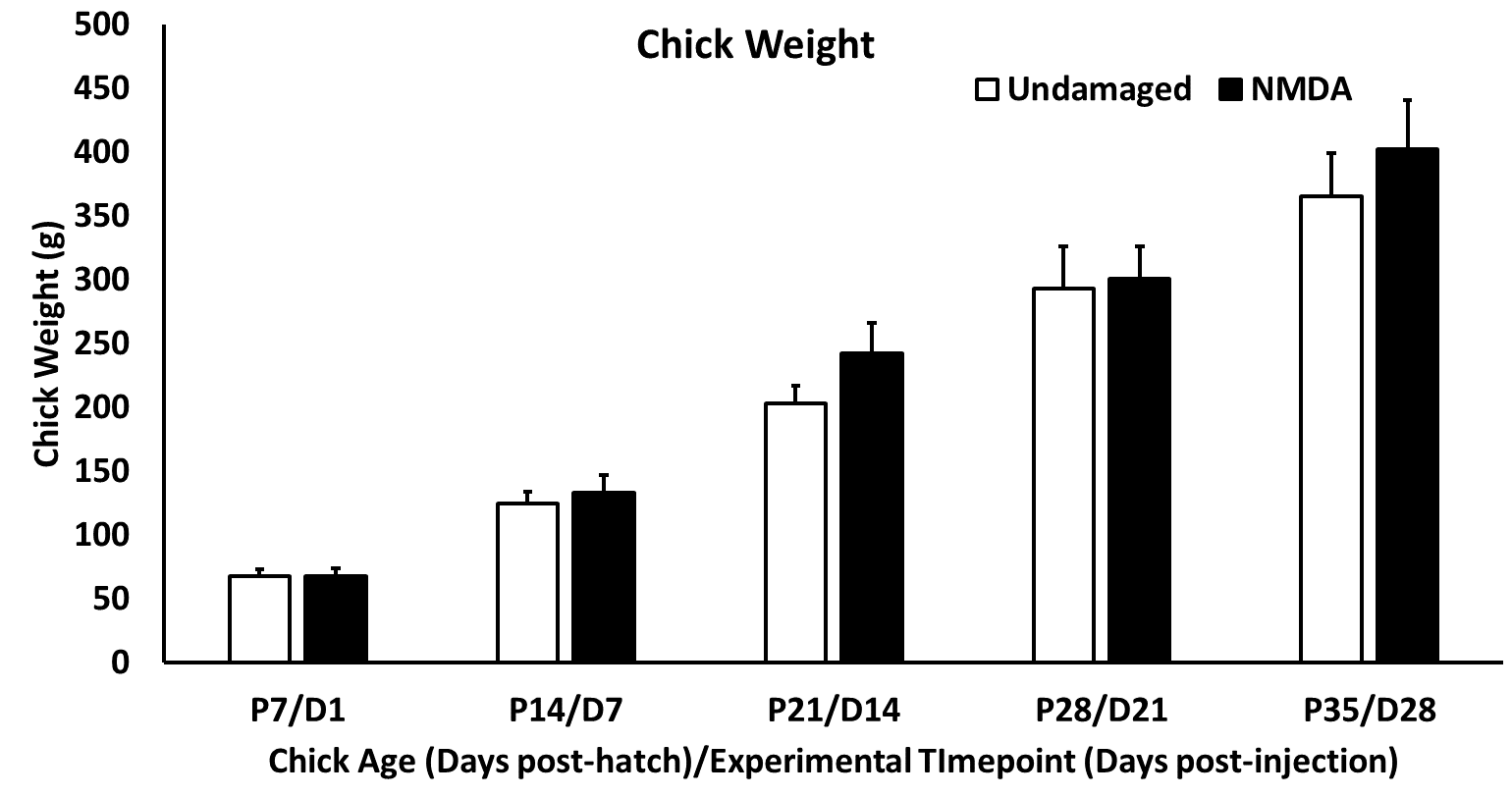

Supplement: S1 Fig — (A) Representative capture of chick retinal layers with the pecten shown in the center. (B) Layer identifiers used by the InVivoView Diver software to measure retinal thicknesses. (C) Representative fundus capture from volumetric OCT scan. Blue crosses represent where measurements were taken. D) Measurements of all retinal thicknesses of the RNFL, IPL, RNFL-IPL, INL, OPL, ONL, ELM, PR, RPE, and full retina layers between NMDA and saline (vehicle) treated eyes at P7/D1 to P35/D28 post injection and their respective age-matched untreated controls. Error bars are shown as standard deviation. Abbreviations: RNFL—retinal nerve fiber layer, GCL—ganglion cell layer, IPL—inner plexiform layer, INL—inner nuclear layer, OPL—outer plexiform layer, ONL—outer nuclear layer, ELM—external limiting membrane, PR—photoreceptors, IS—inner segment, OS—outer segment, ETPRS—end tip of photoreceptors, RPE—retinal pigment epithelium. (TIF) [file pone.0257148.s001.tif]

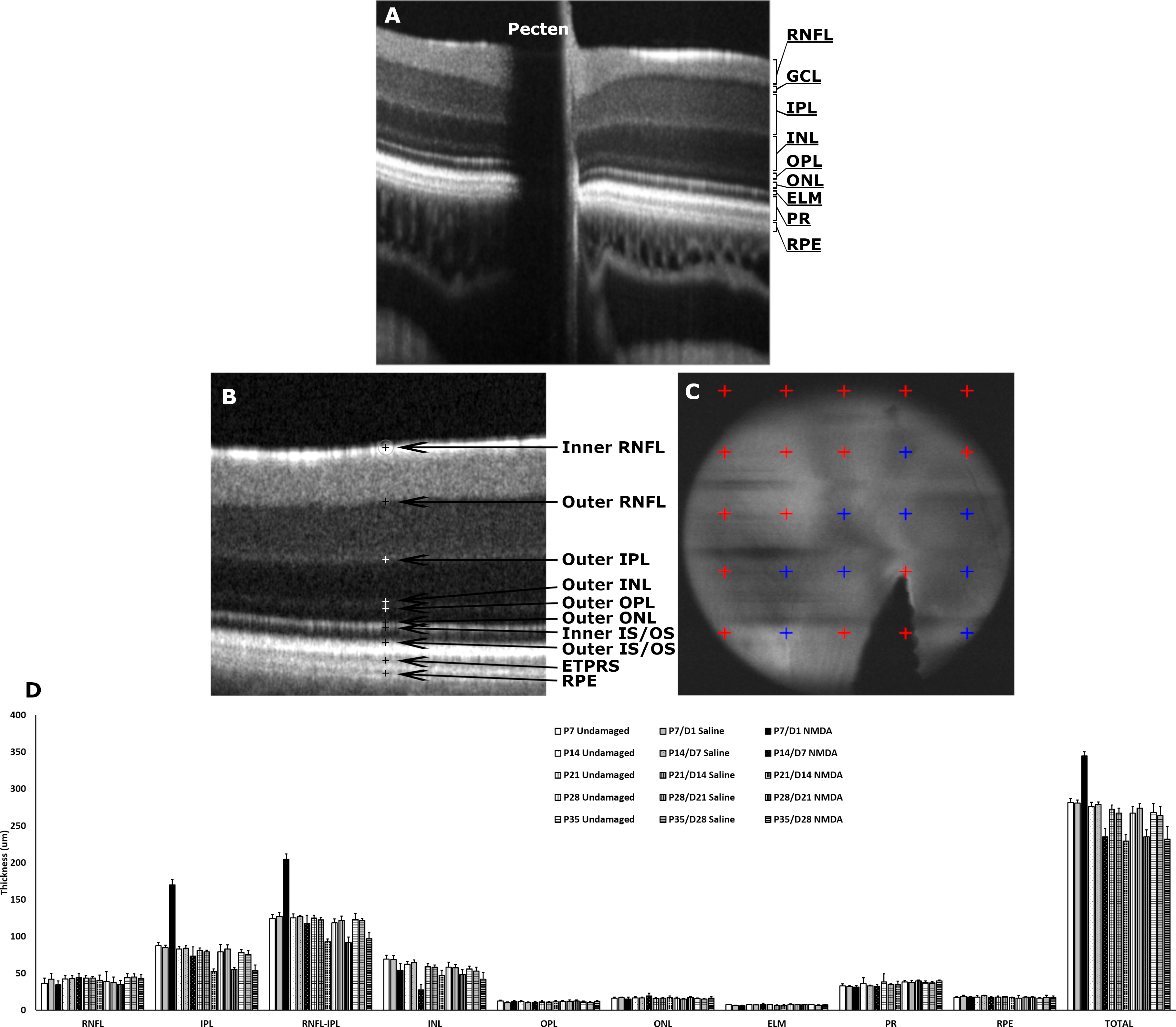

Supplement: S2 Fig — A normative database of undamaged and NMDA-damaged chicks were weighed weekly up until sacrifice at P35. The two treatment groups were typically weighed within one day of each other except for the P21/D14 timepoint where the NMDA-damaged chicks were measured three days after the undamaged chicks due to experimental constraints (n = 10/group). (TIF) [file pone.0257148.s002.tif]

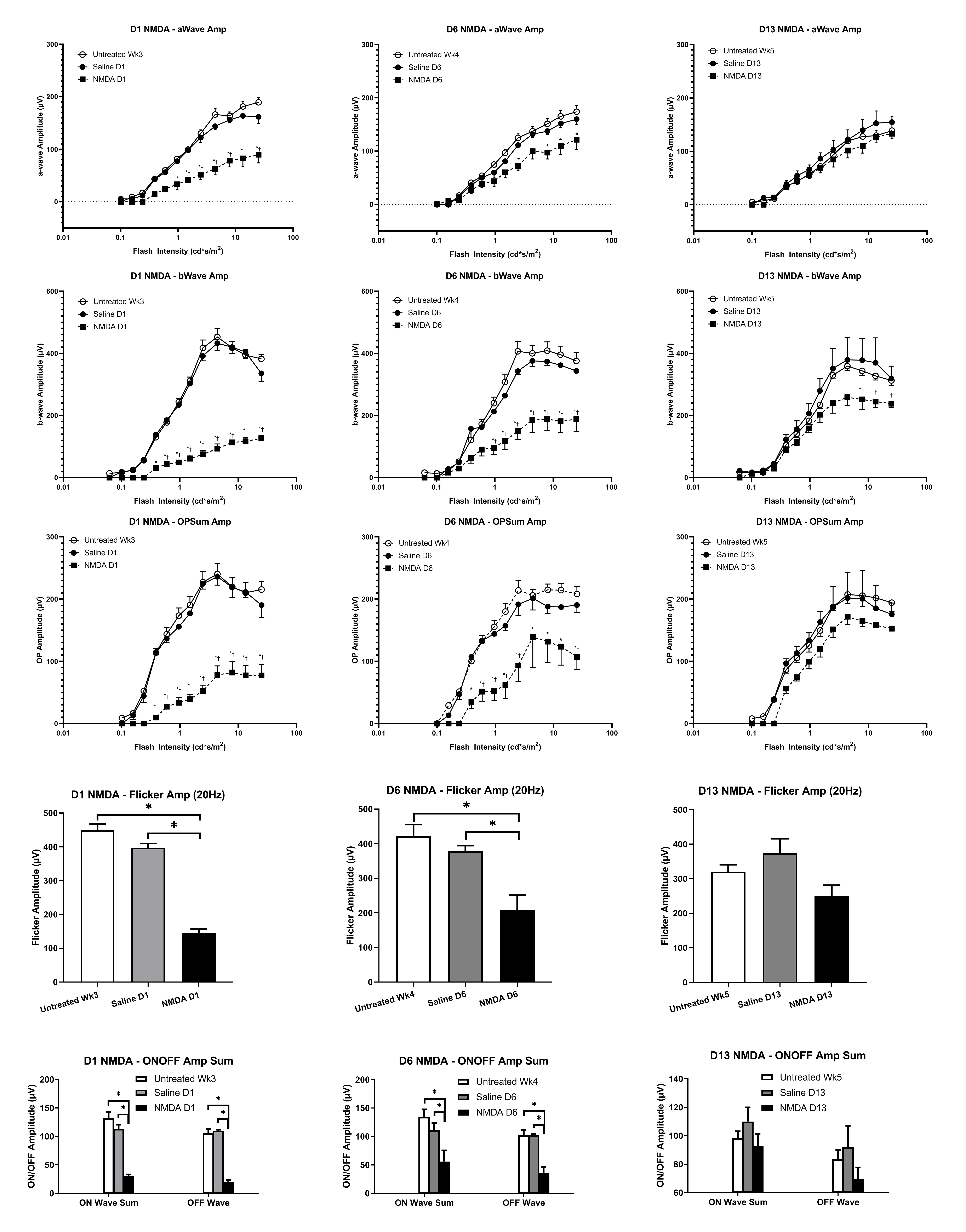

Supplement: S3 Fig — Undamaged chicks (n = 3), NMDA-damaged chick eyes (OS), and saline vehicle-injected fellow chick eyes (OD) (n = 3) were examined with the In Vivo ERG UTAS System. The data is organized by time (columns) and parameter (rows). From left to right, the columns represent one day post-injection (D1) and 3 weeks post-hatch (PWk3), D6/PWk4, and D13/PWk5. From top to bottom, the rows represent a-wave amplitudes, b-wave amplitudes, the amplitudes of the sum of oscillatory potentials (OPSum), flicker amplitudes at 20Hz, and ON/OFF bipolar cell response amplitudes. * indicates significance with vehicle (saline) controls, † indicates significance with undamaged controls. (TIF) [file pone.0257148.s003.tif]

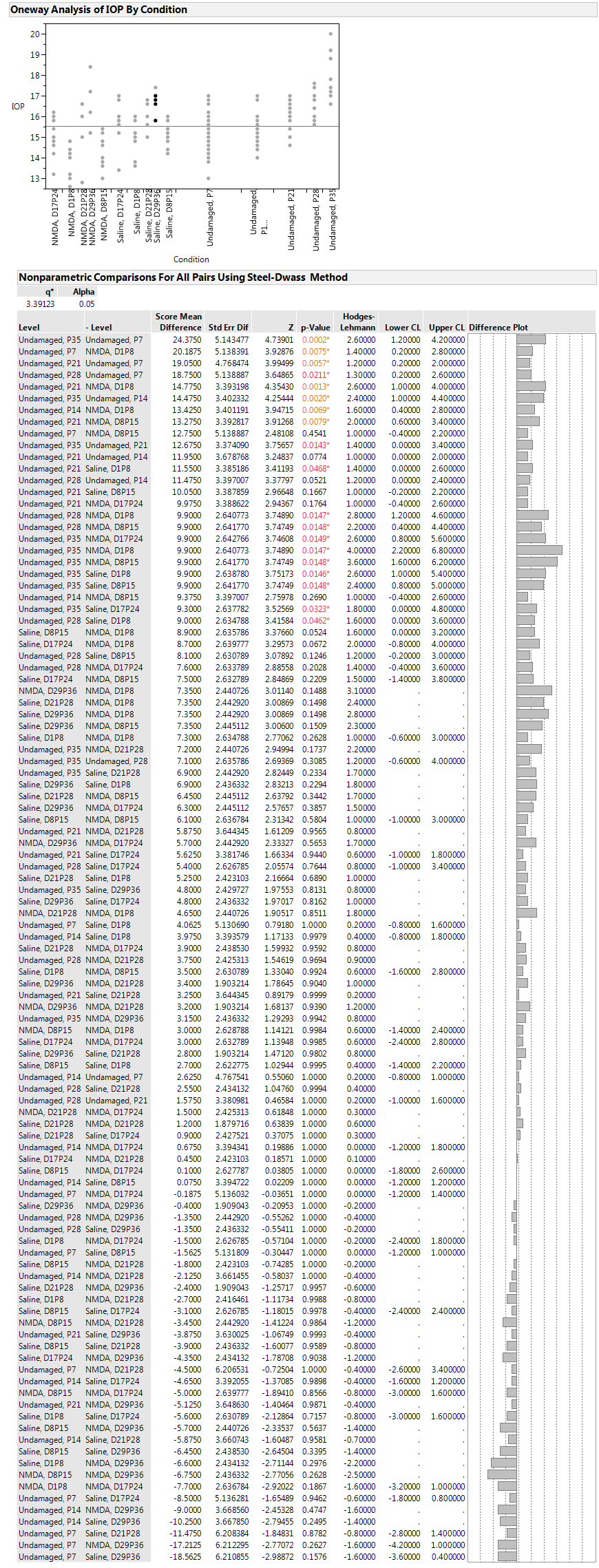

Supplement: S1 File — (ZIP) [file pone.0257148.s004.zip › IOP&Weight/Celeris Chicks, IOP, Nonparametric.png]

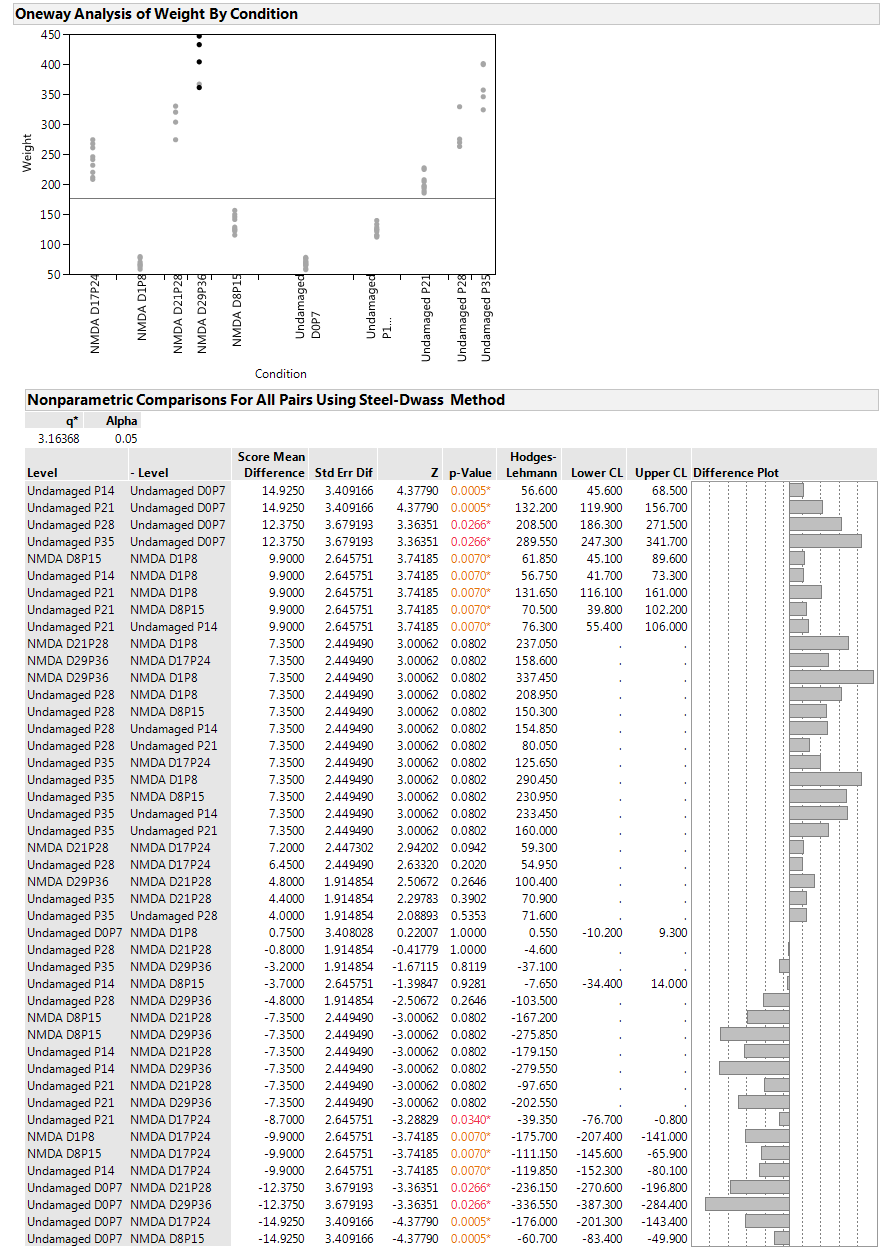

Supplement: S1 File — (ZIP) [file pone.0257148.s004.zip › IOP&Weight/Celeris Chicks, Weight, Nonparametric.png]

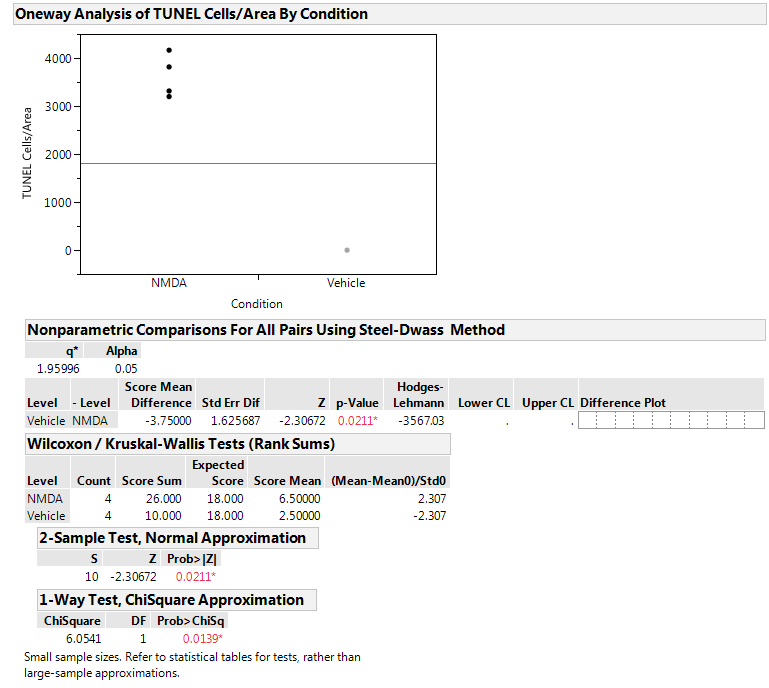

Supplement: S2 File — (ZIP) [file pone.0257148.s005.zip › TUNEL/TUNEL Stats, Non-parametric.png]

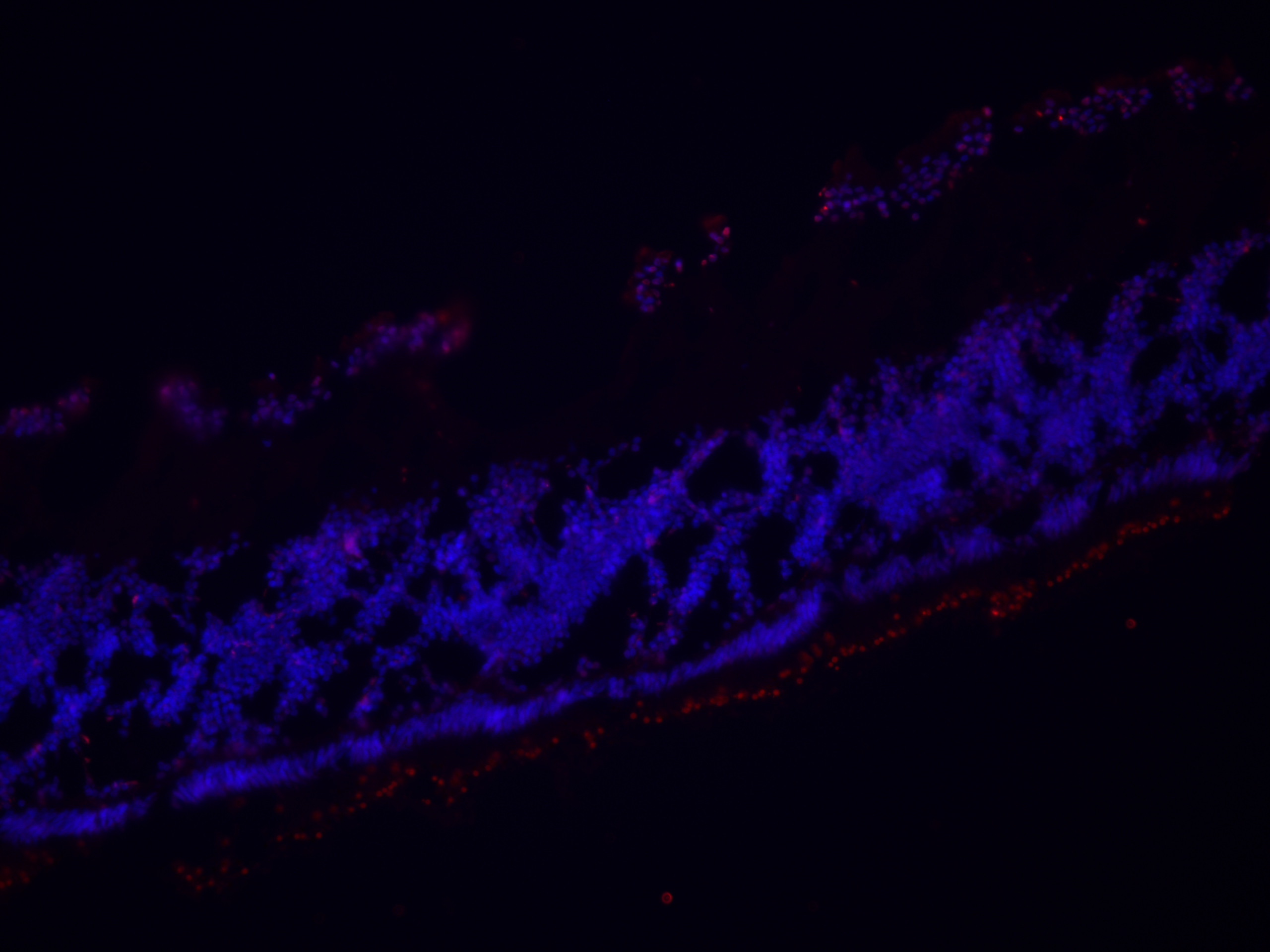

Supplement: S2 File — (ZIP) [file pone.0257148.s005.zip › TUNEL/Untreated/C190408-001/image0144 Merge.tif]

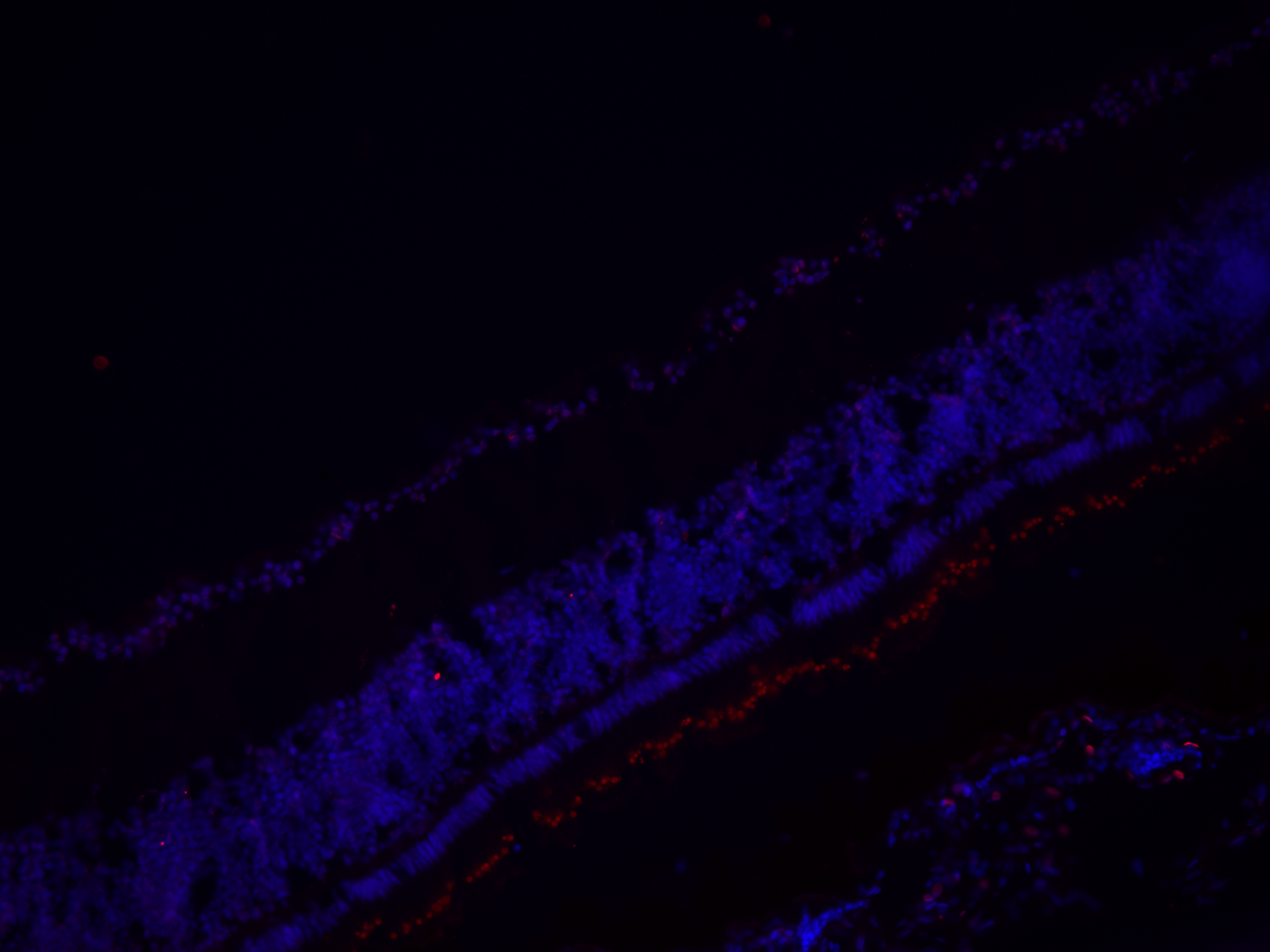

Supplement: S2 File — (ZIP) [file pone.0257148.s005.zip › TUNEL/Untreated/C190408-001/image0146 Merge.tif]

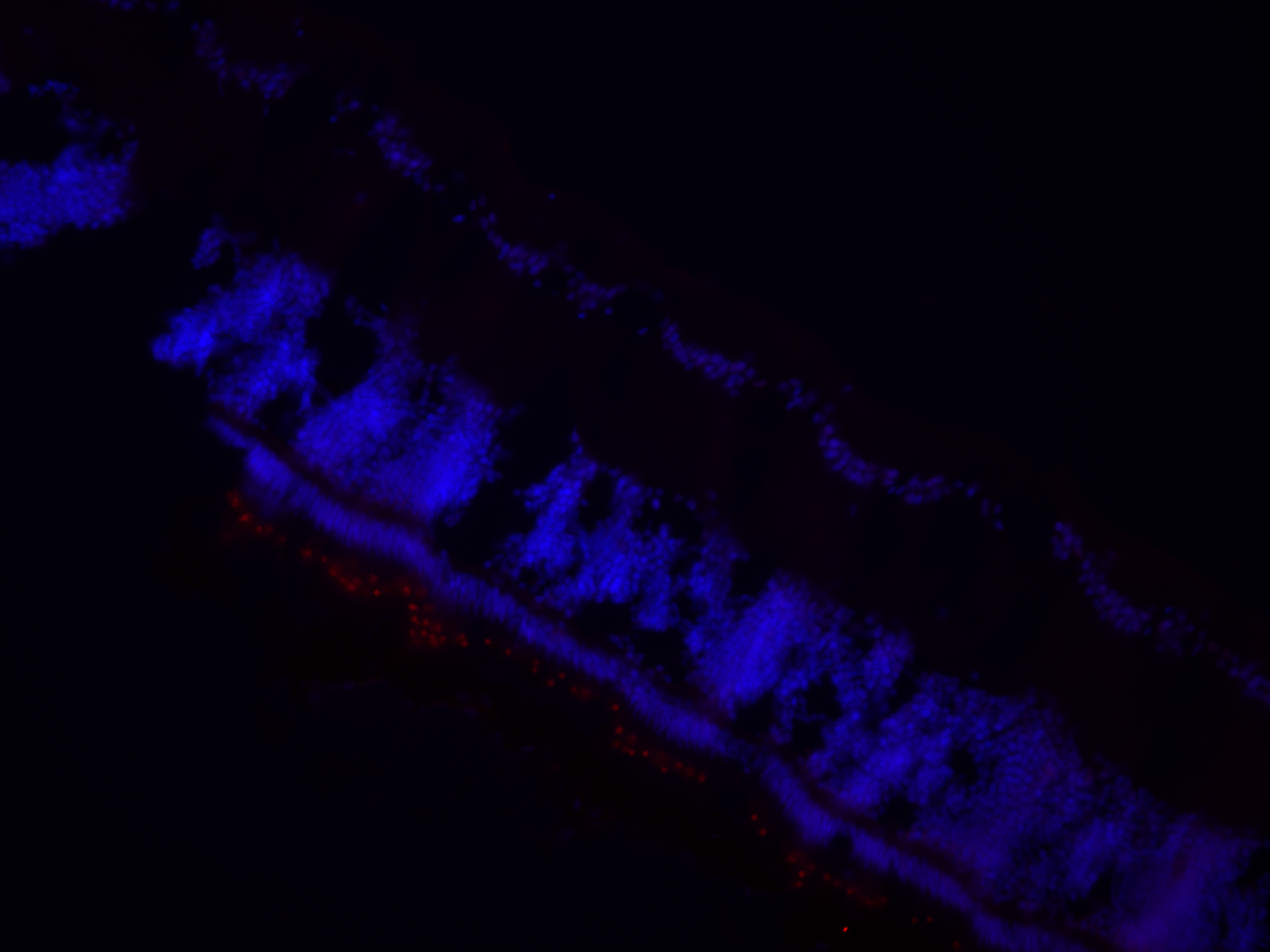

Supplement: S2 File — (ZIP) [file pone.0257148.s005.zip › TUNEL/Untreated/C190408-002/image0148 Merge.tif]

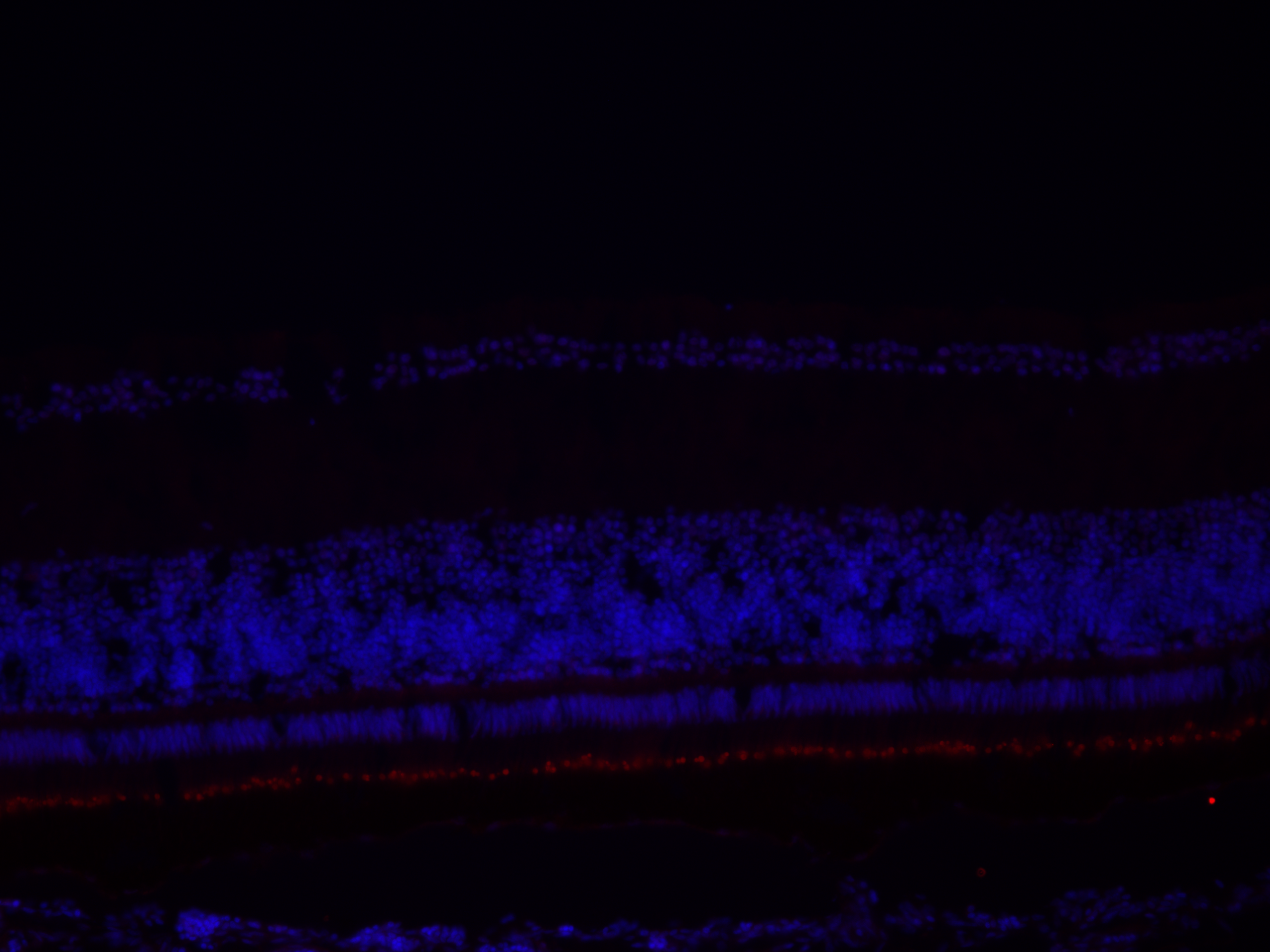

Supplement: S2 File — (ZIP) [file pone.0257148.s005.zip › TUNEL/Untreated/C190408-002/image0150 Merge.tif]

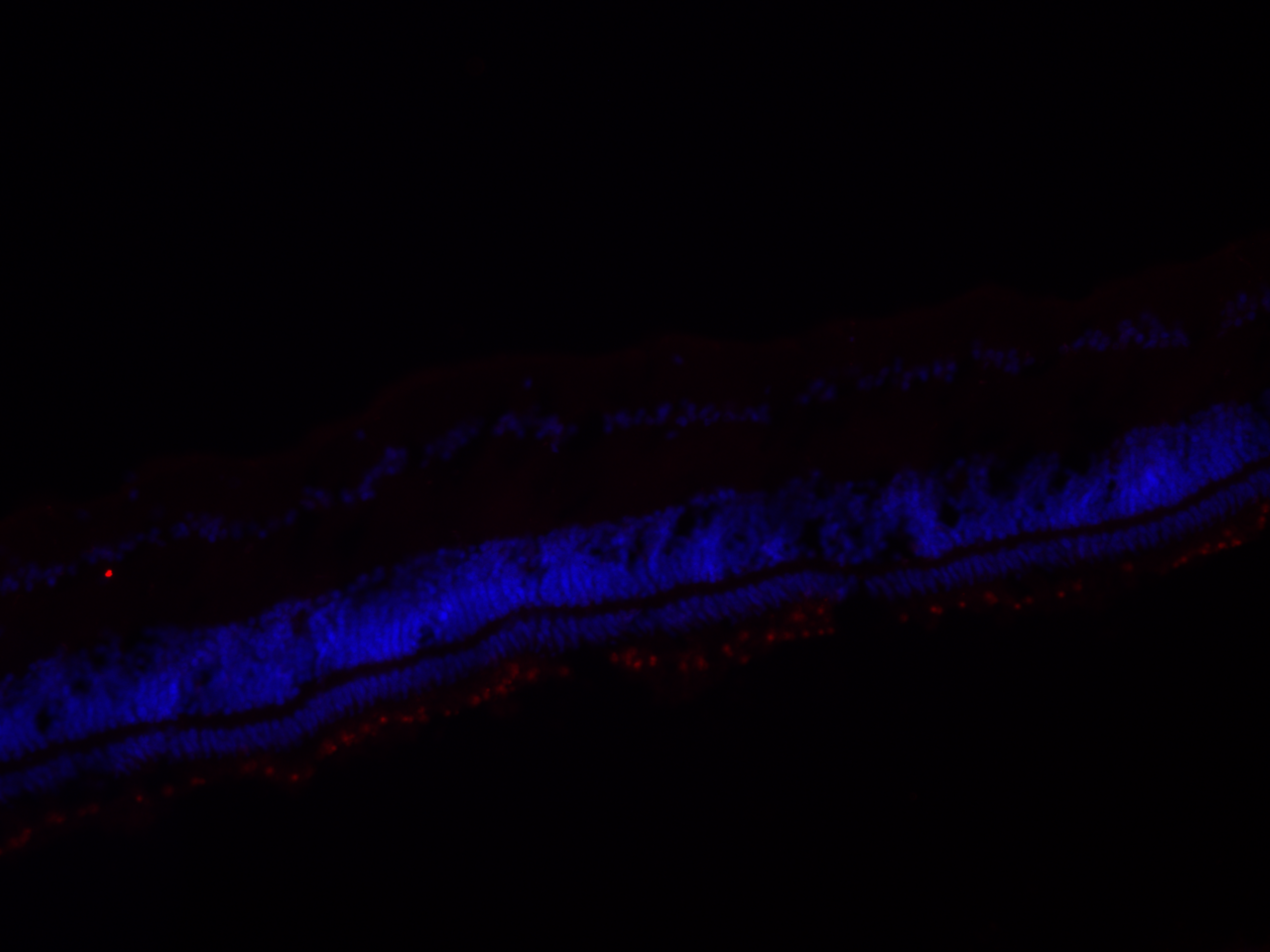

Supplement: S2 File — (ZIP) [file pone.0257148.s005.zip › TUNEL/Untreated/C190408-003/image0152 Merge.tif]

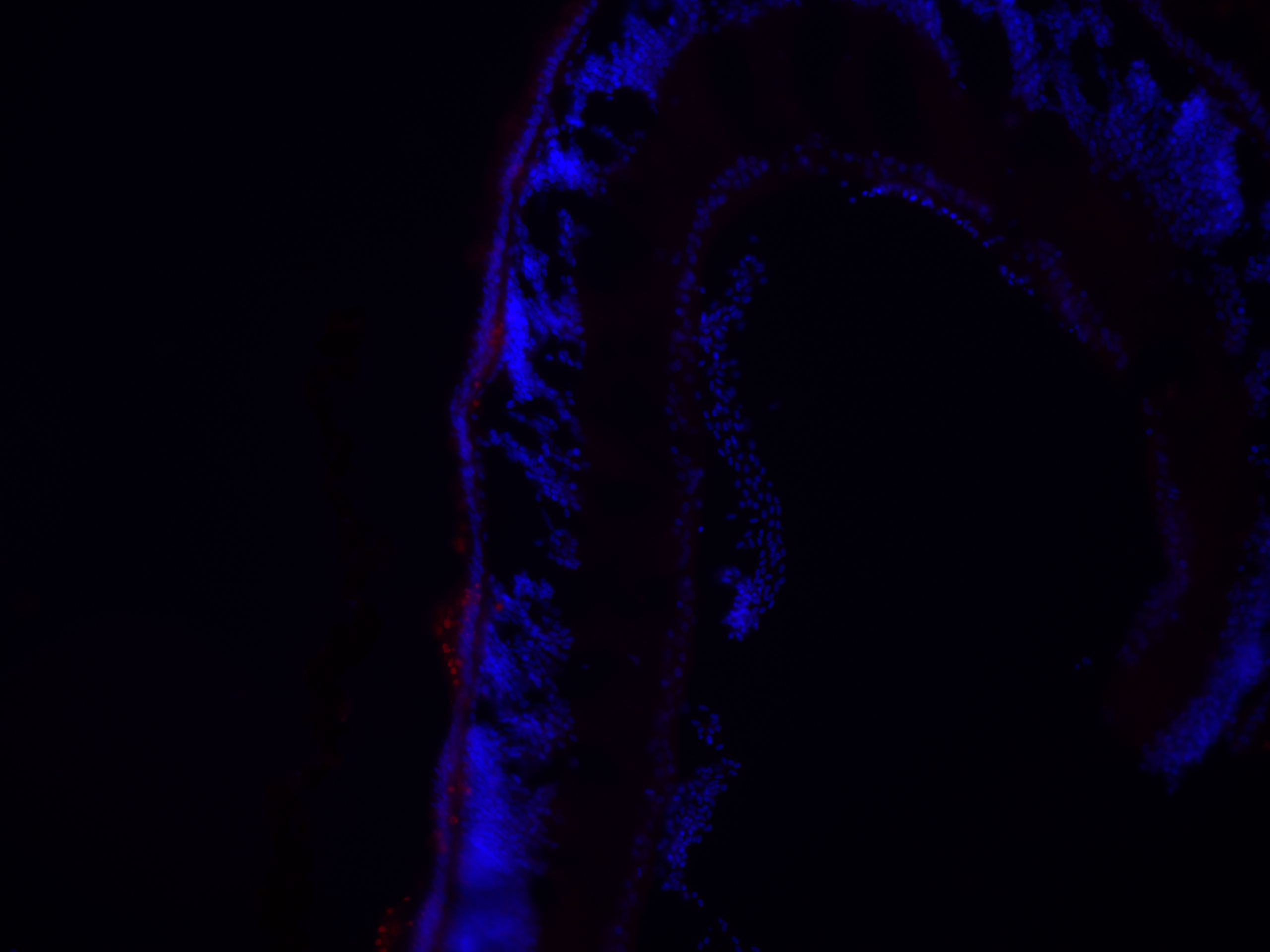

Supplement: S2 File — (ZIP) [file pone.0257148.s005.zip › TUNEL/Untreated/C190408-003/image0154 Merge.tif]

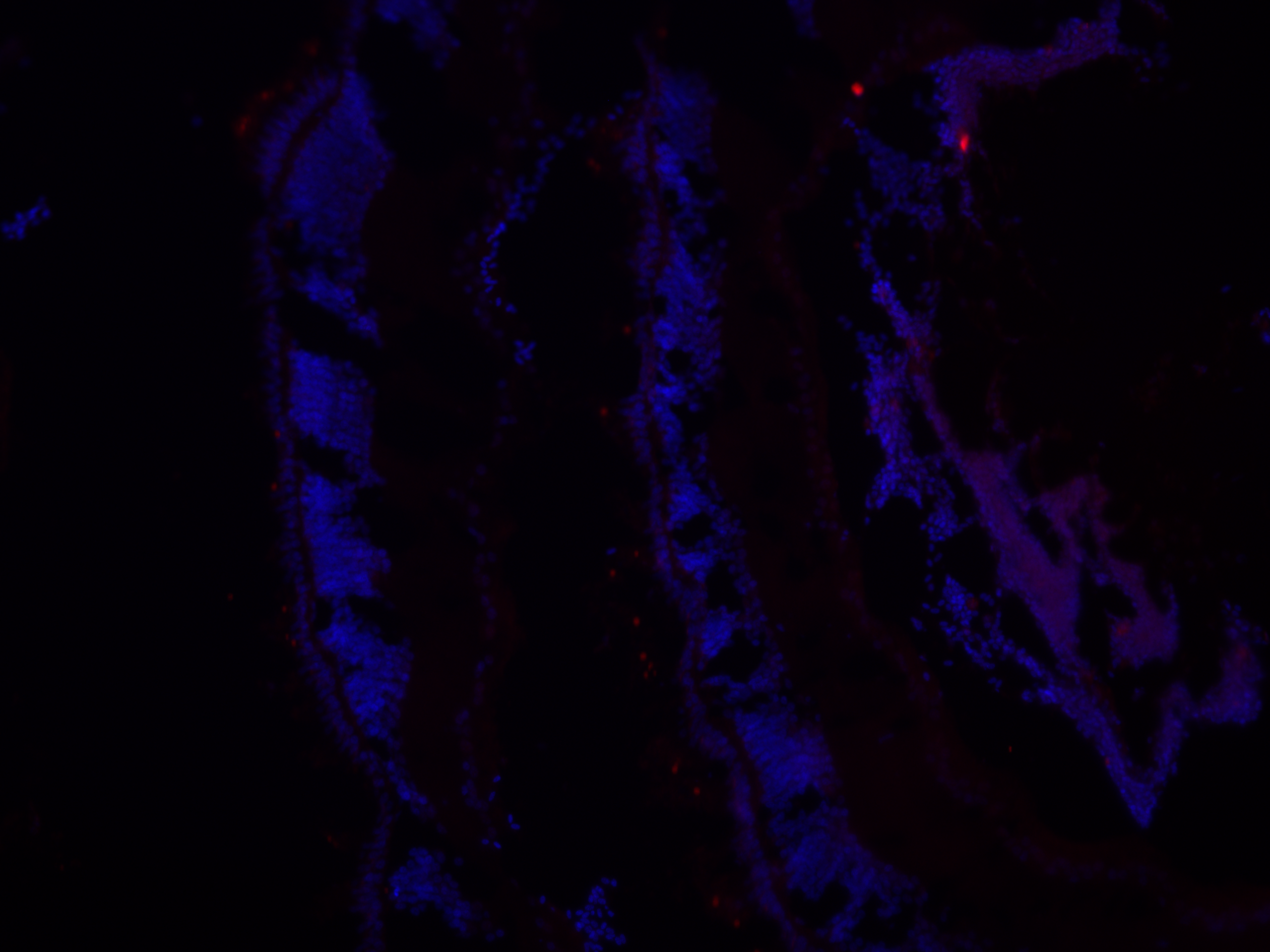

Supplement: S2 File — (ZIP) [file pone.0257148.s005.zip › TUNEL/Untreated/C190408-004/image0156 Merge.tif]

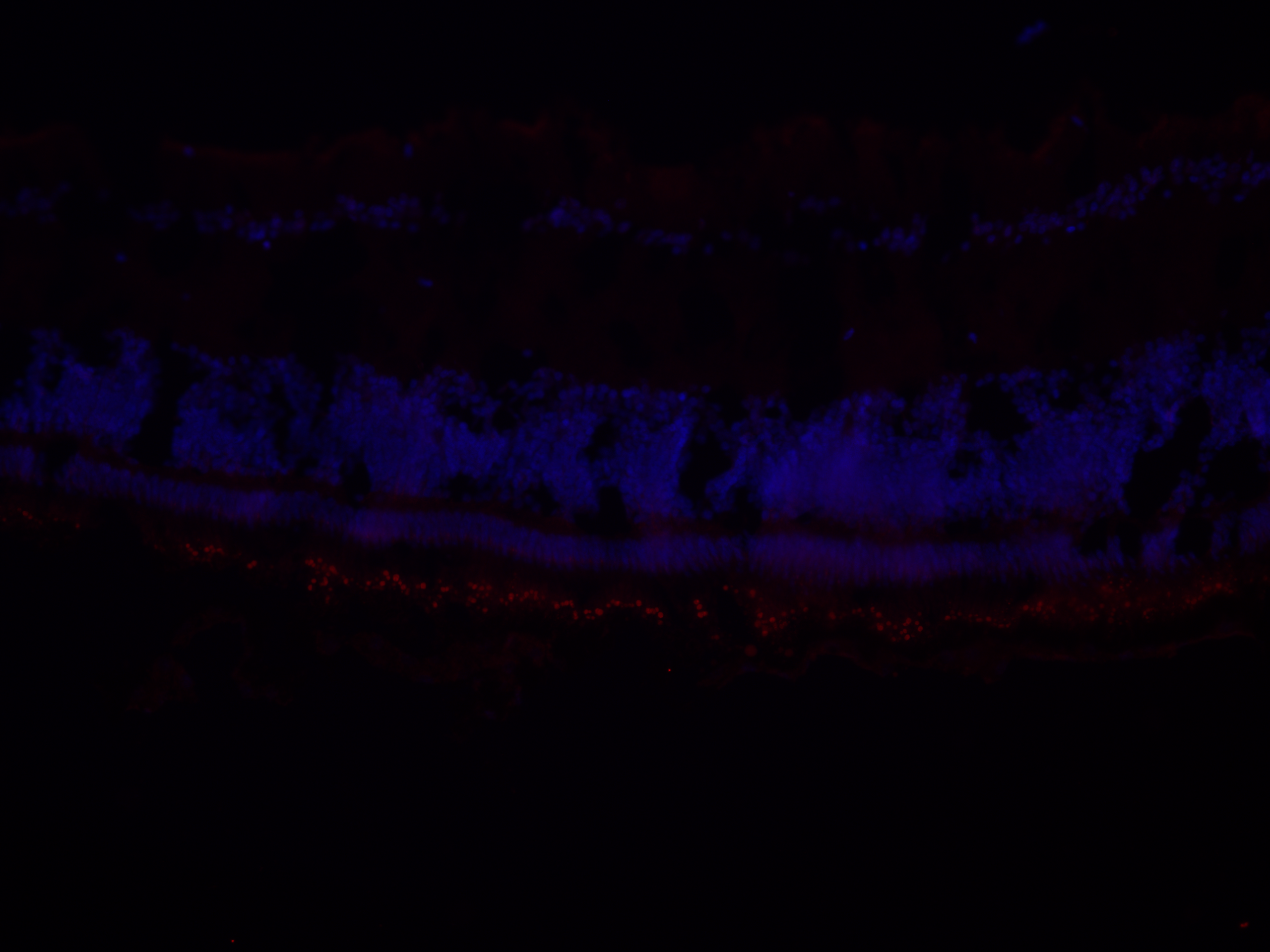

Supplement: S2 File — (ZIP) [file pone.0257148.s005.zip › TUNEL/Untreated/C190408-004/image0158 Merge.tif]

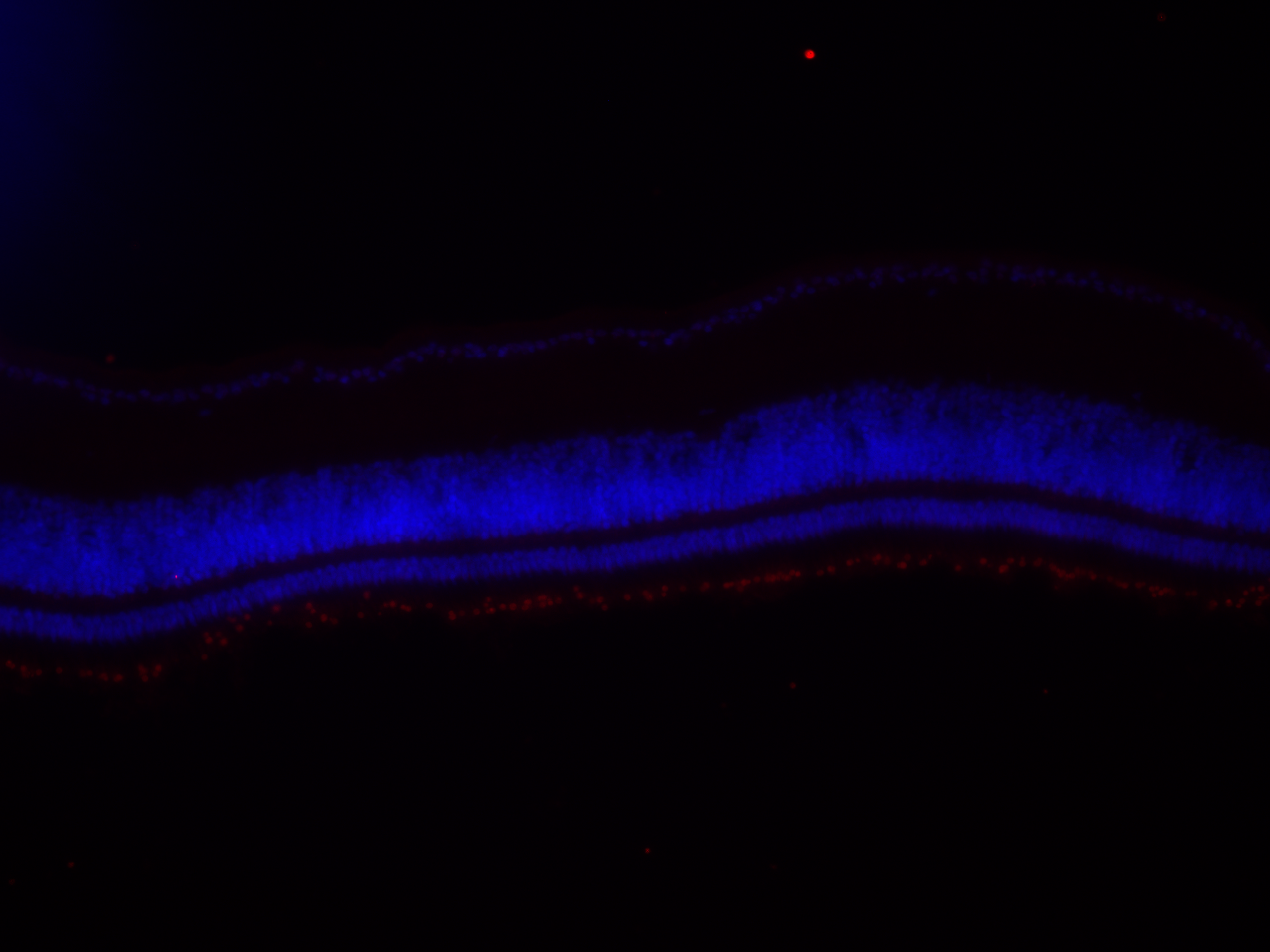

Supplement: S2 File — (ZIP) [file pone.0257148.s005.zip › TUNEL/Vehicle Treated/C190408-005/image0160 Merge.tif]

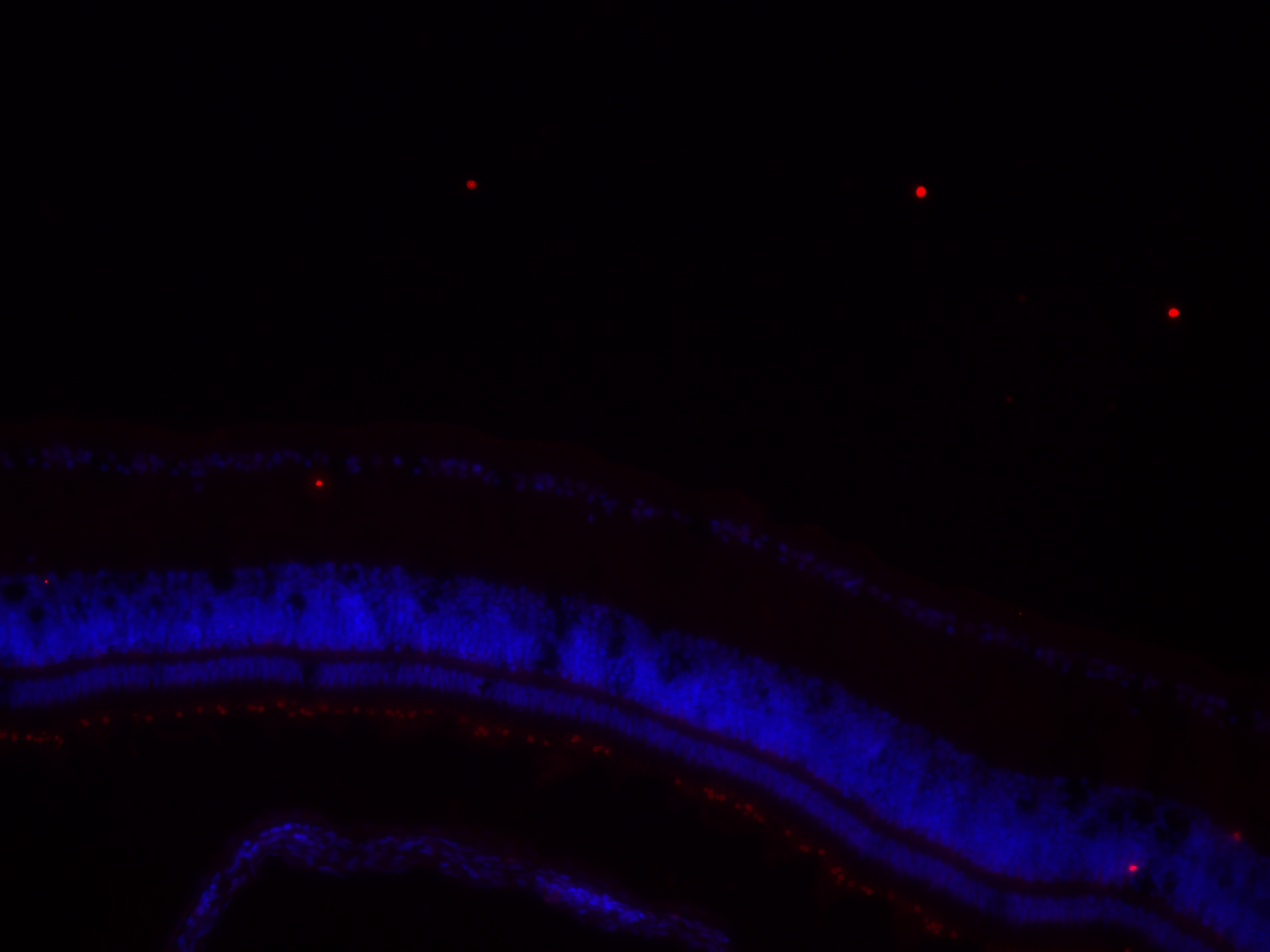

Supplement: S2 File — (ZIP) [file pone.0257148.s005.zip › TUNEL/Vehicle Treated/C190408-005/image0162 Merge.tif]

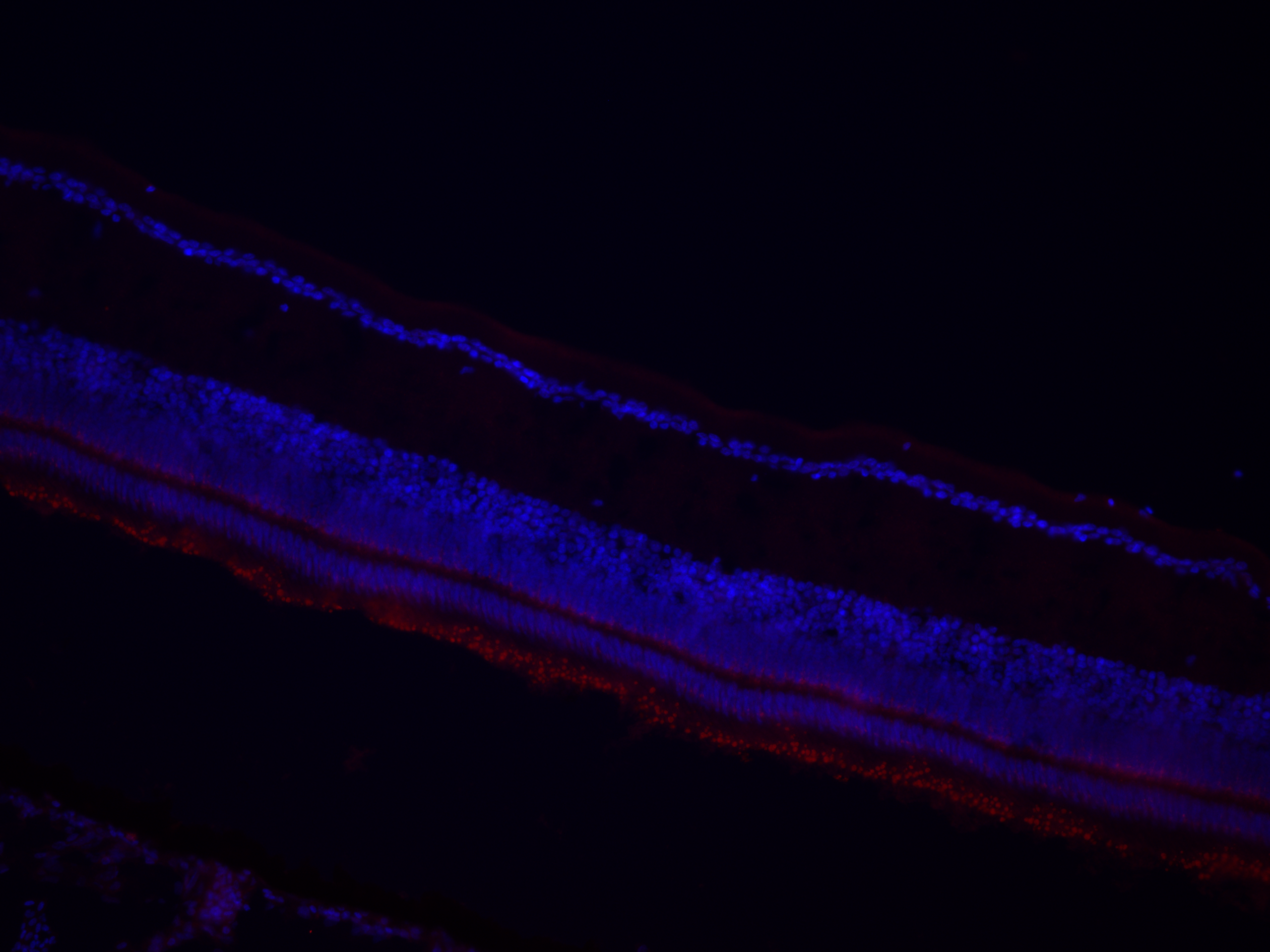

Supplement: S2 File — (ZIP) [file pone.0257148.s005.zip › TUNEL/Vehicle Treated/C190408-006/image0164 Merge.tif]

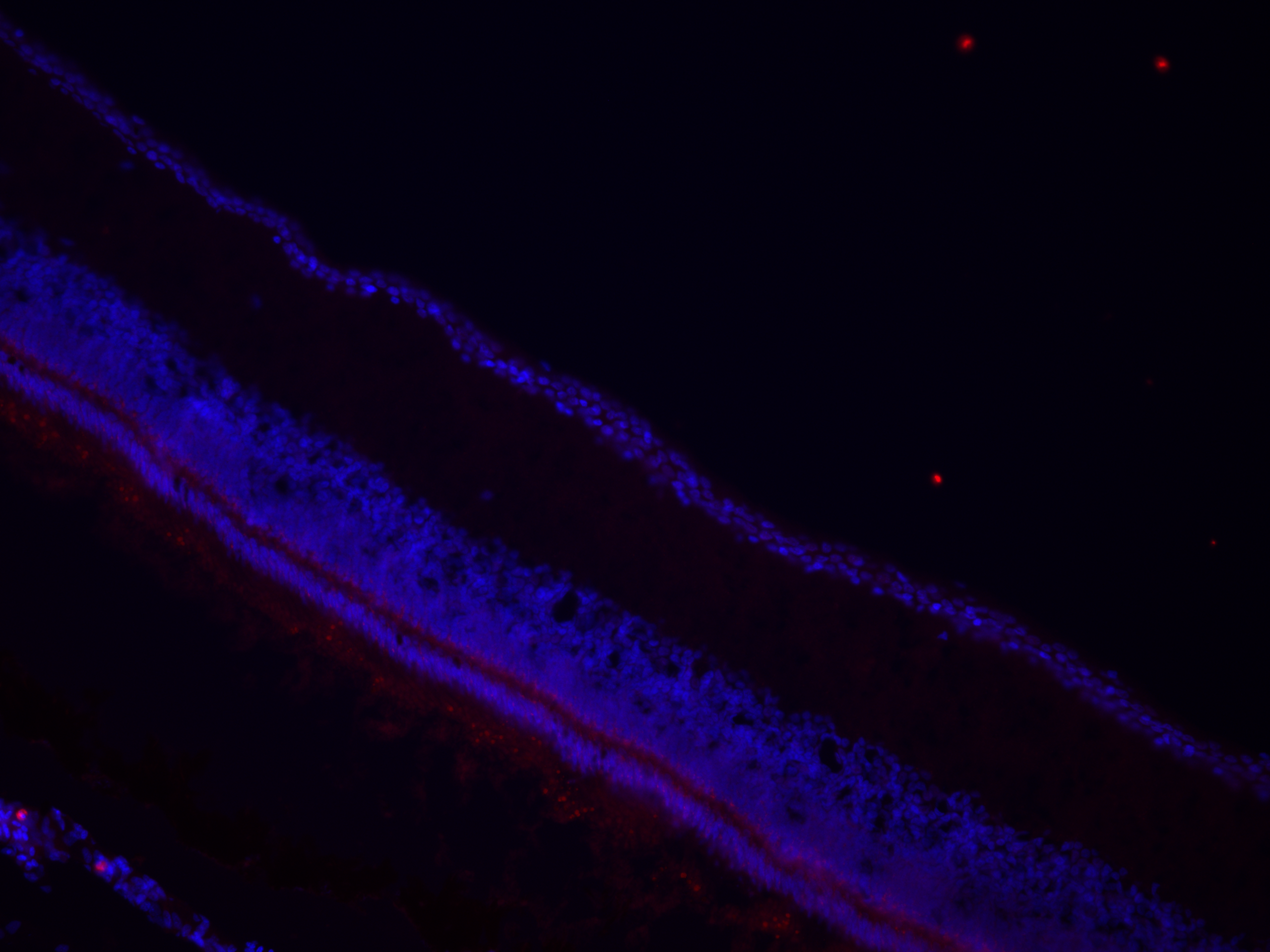

Supplement: S2 File — (ZIP) [file pone.0257148.s005.zip › TUNEL/Vehicle Treated/C190408-006/image0166 Merge.tif]

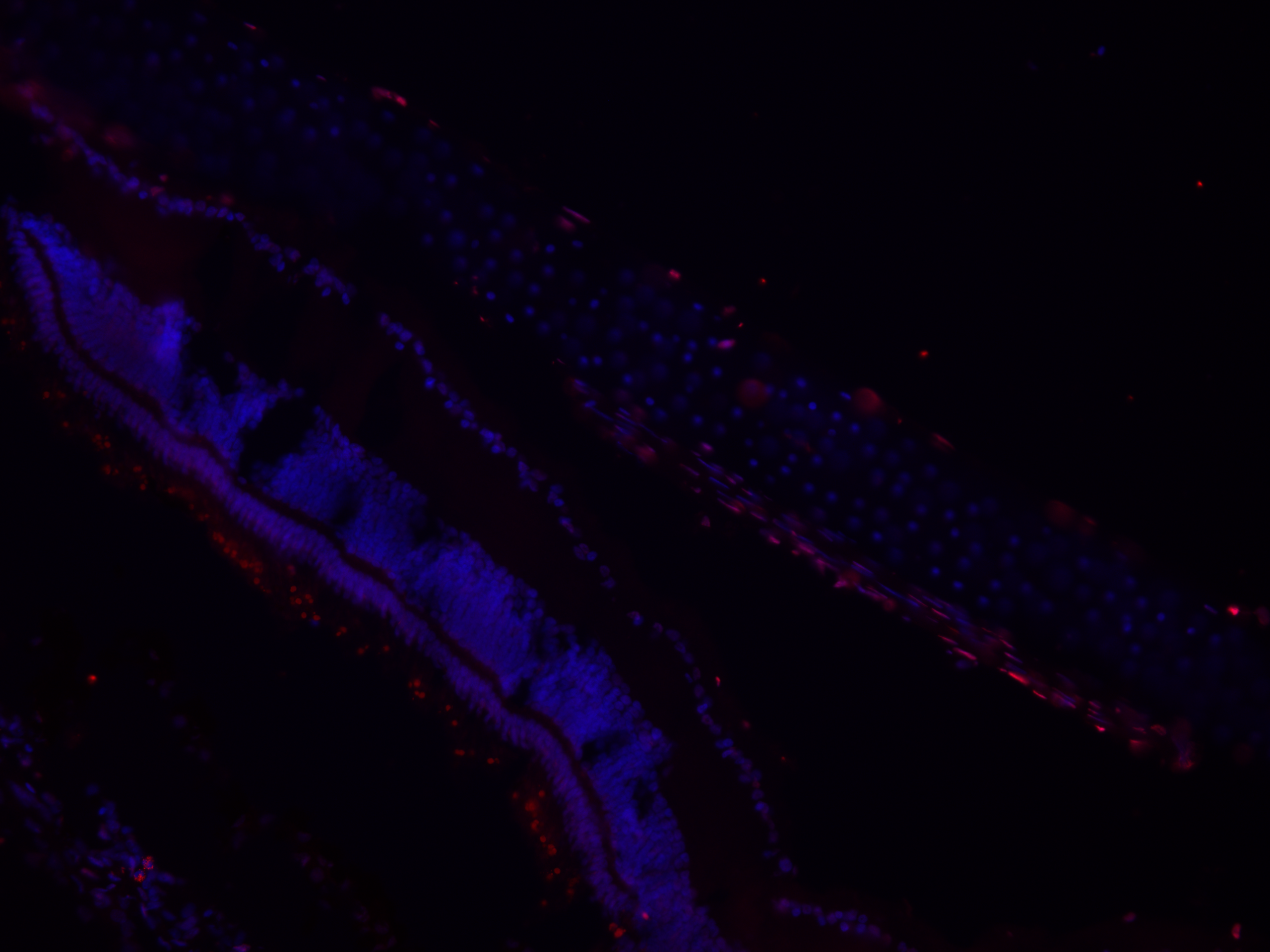

Supplement: S2 File — (ZIP) [file pone.0257148.s005.zip › TUNEL/Vehicle Treated/C190408-007/image0168 Merge.tif]

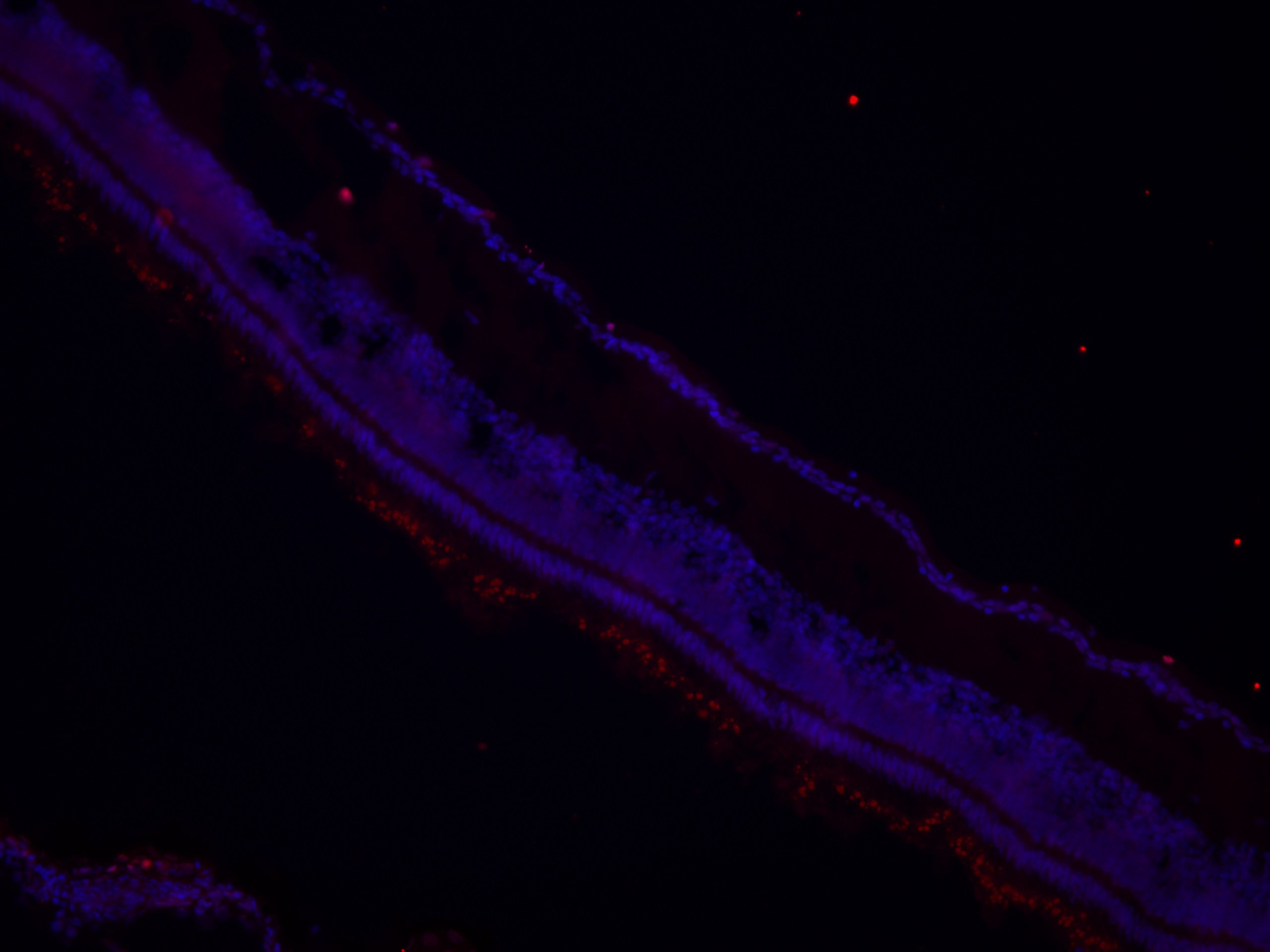

Supplement: S2 File — (ZIP) [file pone.0257148.s005.zip › TUNEL/Vehicle Treated/C190408-007/image0170 Merge.tif]

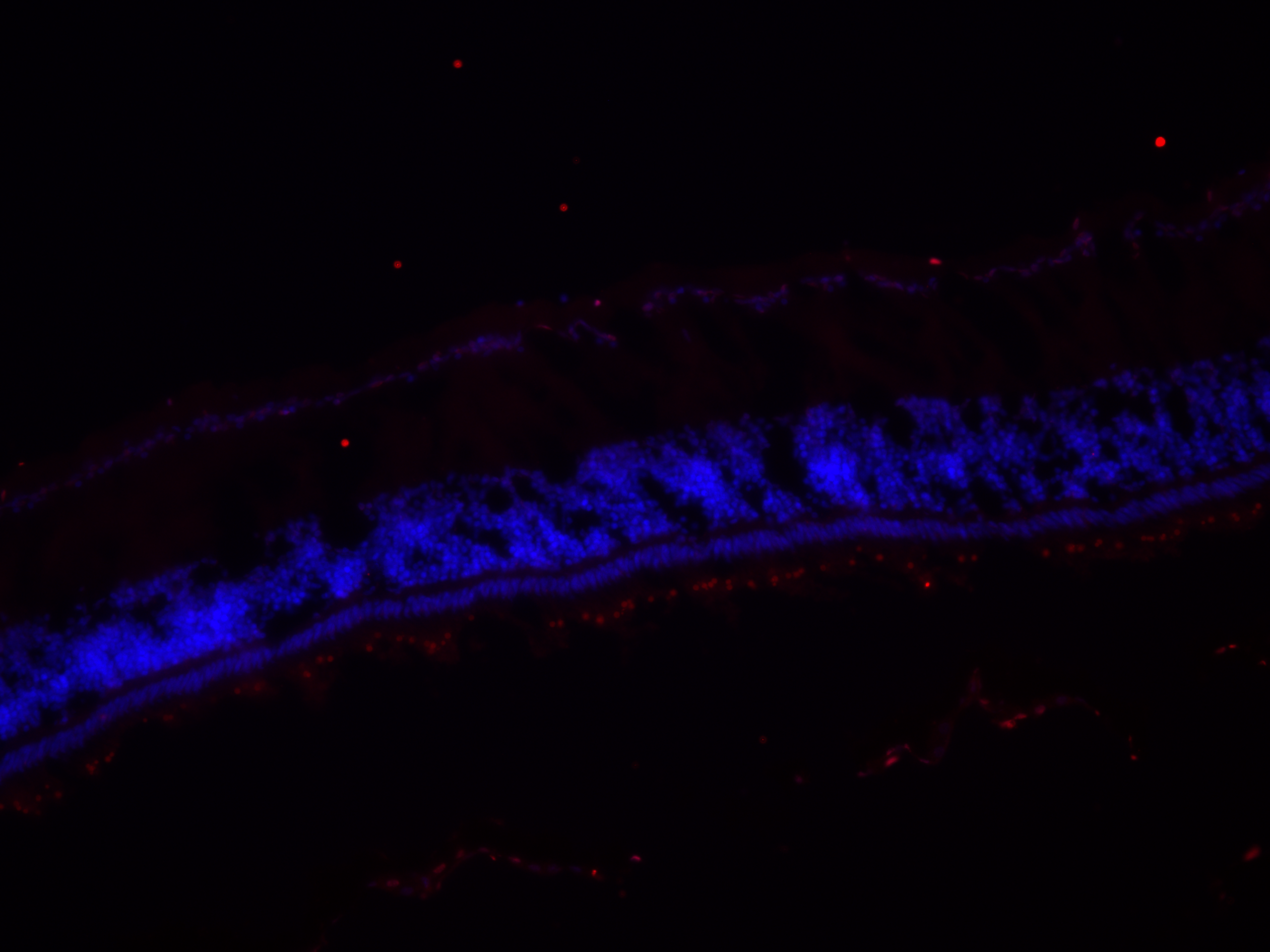

Supplement: S2 File — (ZIP) [file pone.0257148.s005.zip › TUNEL/Vehicle Treated/C190408-008/image0172 Merge.tif]

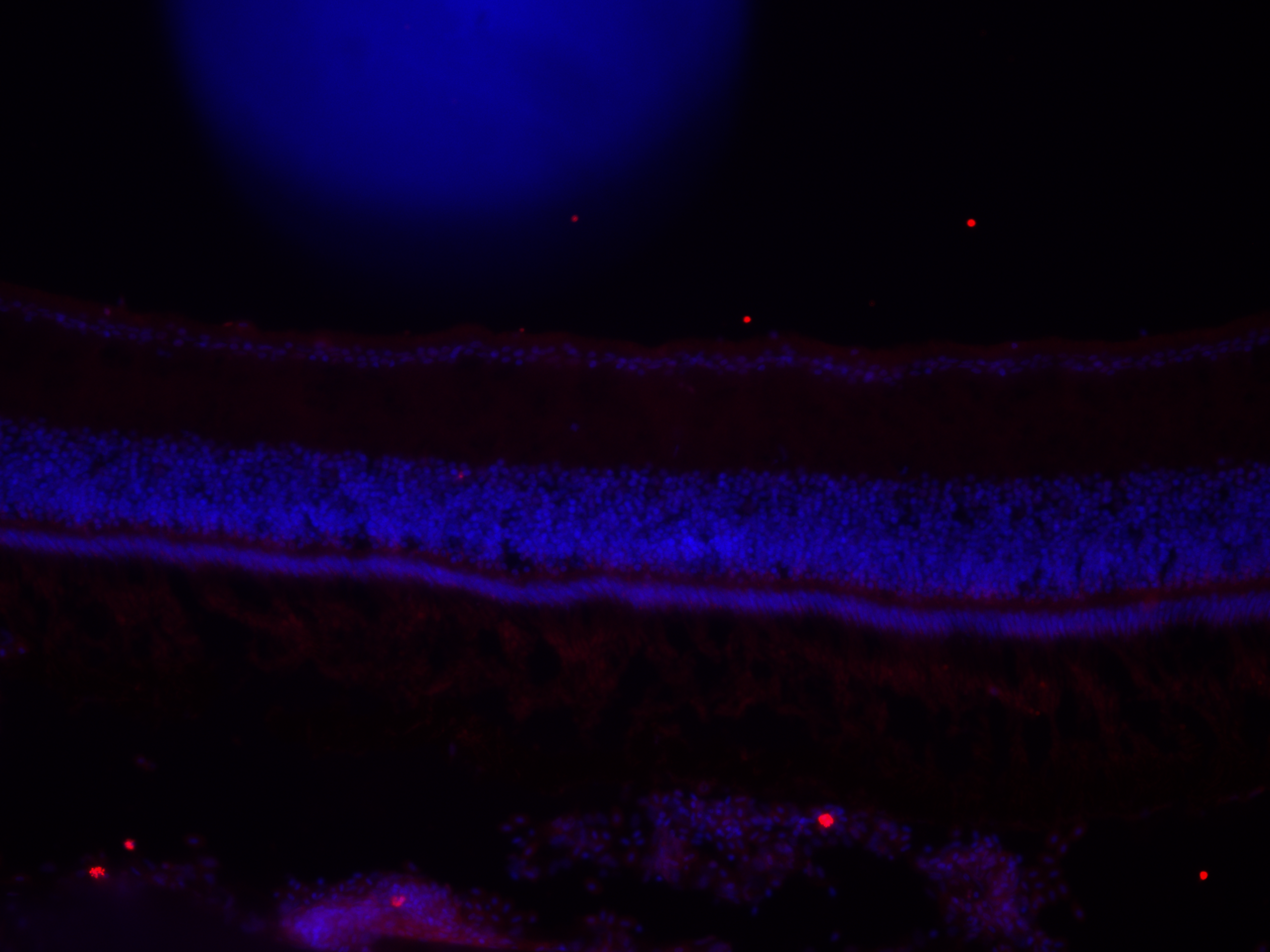

Supplement: S2 File — (ZIP) [file pone.0257148.s005.zip › TUNEL/Vehicle Treated/C190408-008/image0174 Merge.tif]
