# Supplementary material for: Multimodal imaging and functional analysis of the chick NMDA retinal damage model
Source: PLoS One. 2021 Sep 7;16(9):e0257148. doi: 10.1371/journal.pone.0257148 (PMC8423281; doi:10.1371/journal.pone.0257148)
Supplement: S1 File — (ZIP) [file pone.0257148.s004.zip › SD-OCT/C200727/005/D15-PWk3/C200727-005_OS_R_14_0_REGAVG0000085/Manual_PreDefineThreeByFourGridRadialRetina_1.pdf]

Three By Four Grid Radial Retina Template Report

Patient ID	C200727	Name	See InVivoVue
Exam Date	See InVivoVue	Exam Time	See InVivoVue
Eye	OS	Averaged	N/A
Depth (mm)	1.65	Depth Samples	1024
Pitch (um/pix)	14.000 x 2333.333 x 1.608	Refractive Index	See InVivoVue
Scan Type	Radial 14.000mm x 14.000mm x 1.647mm ,<br>1000 x 6 x 1024		

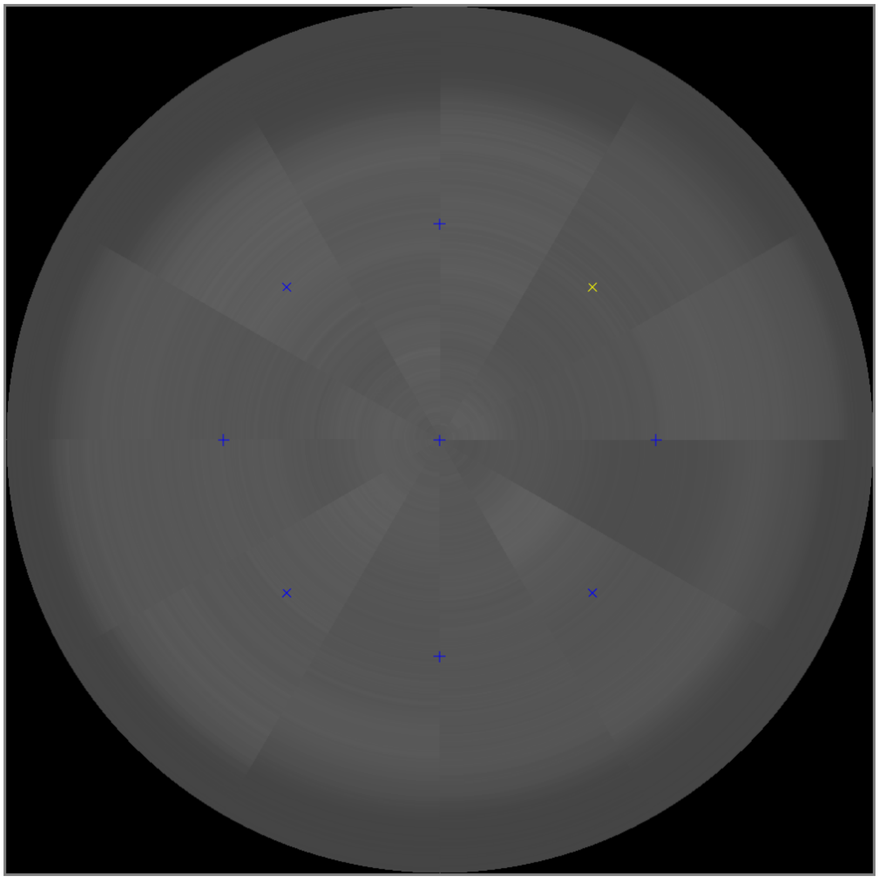

Thickness Metrics

Analysis	Average(mm)	Minimum(mm)	Maximum(mm)	STDEV(mm)
IRNFL - RPE	0.2162	0.2022	0.2265	0.0085
IRNFL - ORNFL	0.0383	0.0231	0.0593	0.0137
ORNFL - OPL	0.1027	0.0793	0.1204	0.0157
OPL - ETPRS	0.0584	0.0549	0.0618	0.0027
